# Evaluating target silencing by short hairpin RNA mediated by the group I intron in cultured mammalian cells

**DOI:** 10.1186/1472-6750-11-79

**Published:** 2011-07-25

**Authors:** Kousei Noguchi, Yoshio Ishitu, Hiroshi Takaku

**Affiliations:** 1 Department of Life and Environmental Science, Chiba Institute of Technology, 2-17-1 Tsudanuma, Narashino, Chiba 275-0016, Japan

## Abstract

**Background:**

The group I intron, a ribozyme that catalyzes its own splicing reactions in the absence of proteins *in vitro*, is a potential target for rational engineering and attracted our interest due to its potential utility in gene repair using trans-splicing. However, the ribozyme activity of a group I intron appears to be facilitated by RNA chaperones *in vivo*; therefore, the efficiency of self-splicing could be dependent on the structure around the insert site or the length of the sequence to be inserted. To better understand how ribozyme activity could be modulated in cultured mammalian cells, a group I intron was inserted into a short hairpin RNA (shRNA), and silencing of a reporter gene by the shRNA was estimated to reflect self-splicing activity *in vivo*. In addition, we appended a theophylline-binding aptamer to the ribozyme to investigate any potential effects caused by a trans-effector.

**Results:**

shRNA-expression vectors in which the loop region of the shRNA was interrupted by an intron were constructed to target firefly luciferase mRNA. There was no remarkable toxicity of the shRNA-expression vectors in Cos cells, and the decrease in luciferase activity was measured as an index of the ribozyme splicing activity. In contrast, the expression of the shRNA through intron splicing was completely abolished in 293T cells, although the silencing induced by the shRNA-expressing vector alone was no different from that in the Cos cells. The splicing efficiency of the aptamer-appended intron also had implications for the potential of trans-factors to differentially promote self-splicing among cultured mammalian cells.

**Conclusions:**

Silencing by shRNAs interrupted by a group I intron could be used to monitor self-splicing activity in cultured mammalian cells, and the efficiency of self-splicing appears to be affected by cell-type specific factors, demonstrating the potential effectiveness of a trans-effector.

## Background

The group I intron from *Tetrahymena thermophila *catalyzes its own excision and ligation of the 5' and 3' exons, meaning that it performs self-splicing without the aid of proteins *in vitro *[1]. Self-splicing of unusual sequence alignments has been shown to occur *in vitro *and *in vivo *in other species, including mammalian species, but group I introns have not been reported in mammals [2]. In addition to self-splicing to ligate two exons that are juxtaposed to a group I intron, several variations on self-splicing have been identified, including trans-splicing, which is used to correct gene sequences with mutations, a promising technique with potential therapeutic application [3,4]. Thus, elucidation of the actions of group I introns *in vivo *would be beneficial [5,6].

The proper structure and the efficient catalysis of group I and group II introns seem to depend on RNA chaperone proteins *in vivo *[7]. Without the aid of chaperone proteins, misfolding is often triggered from alternative base pairings or in the thermodynamically favored direction. To investigate the splicing activity of the group I intron in cultured mammalian cells, we constructed expression vectors containing a short hairpin RNA (shRNA) interrupted by an intron. The expressed shRNA is converted to a small interfering RNA (siRNA) by the RNase III enzyme DICER, and then it triggers RNA interference (RNAi) in the cells [8-10]. After splicing with the help of chaperone proteins, the resulting ligated transcript is predicted to fold back on itself to form a stem-loop structure that produces shRNA, leading to silencing of the targeted reporter gene. The low production level of protein products due to self-splicing limits the utility of group I introns and self-splicing, but in this context, the self-splicing mechanism can be used to produce RNA as the final product [2]. Therefore, this method might provide insight into the mechanism of self-splicing and into the relationship between the self-splicing mechanism and RNA chaperone proteins *in vivo*.

Two sets of coaxially stacked helices, P4-P6 and P3-P9, are the conserved core of group I introns, and the peripheral domains play important roles in stabilizing the functional structures. It has been suggested that P5abc stabilizes the folded structure of group I introns, and the interaction between the tetraloop (L5b) in P5abc and the tetraloop receptor in the P6 stem functions as a clamp to stabilize the conformation [11]. A chaperone protein can substantially rescue the catalytic activity of *Tetrahymena *introns in which P5abc has been deleted. This chaperone appears to bind the P4-P6 domain and to form a scaffold for the assembly of the P3-P9 domain [12]. Group I ribozymes were successfully activated using an appended aptamer in which the P6 region of the intron from the thymidylate synthase gene in bacteriophage T4 was substituted with an anti-theophylline aptamer [13]. Here, we also attempted substitution of the P6 region of the *Tetrahymena *intron with an anti-theophylline aptamer so that the specific binding of theophylline to the the group I intron might lead to a conformational change, thus modulating catalysis in a way that is similar to the binding of a chaperone protein. The application of functional RNAs to gene regulation has intriguing possibilities for conditional gene expression and knockdown systems, but the development of these applications is still in progress [14-17].

In the present study, we used silencing of the firefly luciferase gene by shRNAs to evaluate the splicing efficiency of the *Tetrahymena thermophila *group I intron, which was inserted into the loop region, in a mammalian cultured cell line. In addition, to infer how self-splicing was affected by a trans-effector in mammalian cells, we assessed the effects of an appended aptamer under splicing-competent physiologic conditions.

## Results

### Construction of shRNAs containing a microRNA-derived loop and validation of target silencing

**Figure 1:**
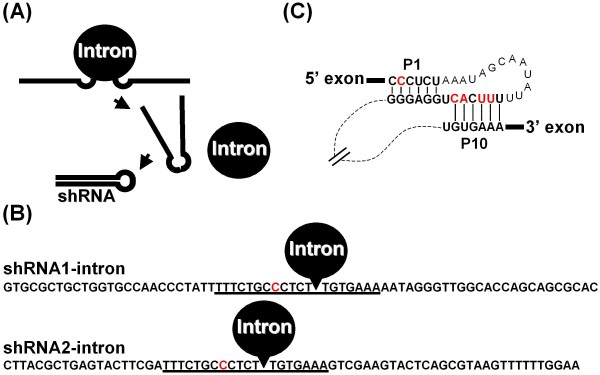
**shRNA directed against firefly luciferase and interruption of shRNA by an intron**. (A) Schematic representation of the formation of the stem-loop structure through the self-splicing of a group I intron inserted into the loop site. (B) Expression vectors were constructed for shRNAs targeting the "155-173" or "851-875" region of firefly luciferase. The shRNA cassettes were placed under control of the CMV promoter of the pRNA-CMV3.1-Neo vector. The loop portion of the shRNA was from the loop region of hsa-mir-371 with a slight modification. The loop sequences are underlined. The inserted site of the group I intron is illustrated, and the modified nucleotides are shown in red. (C) Schematic representation of the interactions involved in self-splicing including the pairing between the intron and the 5' and 3' exon sequences. These pairings, namely the P1 helix (5'exon-intron pairing) and P10 helix (intron-3'exon pairing), are shown in bold, and the modified nucleotides are shown in red.

The production of shRNA via splicing catalyzed by a *Tetrahymena *group I intron estimates the splicing efficiency in conjunction with chaperone proteins in mammalian cells (Figure 1A). To this end, shRNAs targeting the firefly luciferase transcript were placed under a cytomegalovirus (CMV) promoter, and a group I intron was inserted into the loop region (Figure 1B). Then target silencing by the shRNA was quantitatively analyzed as a reflection of the inserted ribozyme activity. Of note, the transcribed RNA was longer than the shRNA and was expected to be fully expressed under these conditions. Specifically, RNA polymerase II-driven promoters have a variety of expression patterns and are more suitable for achieving a spatio-temporal expression pattern than RNA polymerase III-driven promoters [18]. The sequences of the 5' and 3' exons of the *Tetrahymena *intron were designed to pair with the intron for efficient and precise splicing, namely the formation of the P1 helix (5' exon-intron pairing) and the formation of the P10 helix (intron-3' exon pairing). To accommodate these characteristics, we searched for a loop portion that was similar to the pairing sequences using a microRNA (miRNA) database, and the hsa-mir-371 sequence with a one-base modification was selected [19]. The one base change of the loop region did not significantly decrease the efficacy of silencing by the shRNA (data not shown). In addition, 4 bases of the intron sequence were modified in conjunction with the loop (Figure 1B, C).

**Figure 2:**
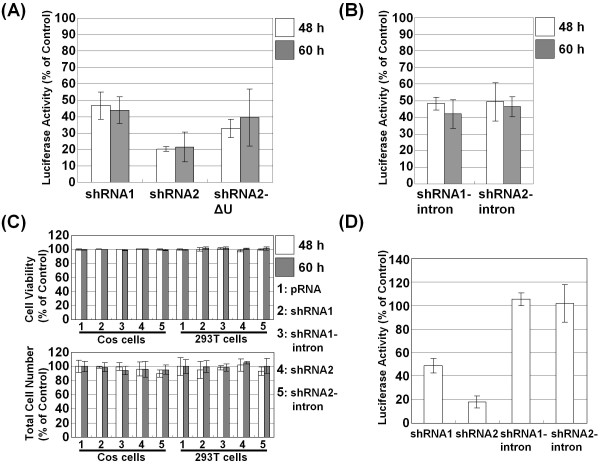
**Characterization of shRNA and shRNA interrupted by an intron in cultured cells**. (A) The efficiency of the shRNA was analyzed by transiently transfecting Cos cells with vectors expressing firefly luciferase and *Renilla *luciferase. Firefly and *Renilla *luciferase activity was analyzed 48 h and 60 h after transfection using the Dual-luciferase Reporter Assay System in which firefly luciferase activity is normalized to *Renilla *luciferase activity. The pRNA-CMV3.1-Neo empty vector was used as a control, and the results are expressed as the mean ± S.D. of the percentage of control. (B) Silencing activity of the shRNA1-intron and shRNA2-intron in Cos cells 48 h and 60 h after transfection. (C) Cell viability was determined microscopically by trypan blue exclusion 48 h and 60 h after transfection with the pRNA-CMV3.1-Neo, shRNA1, shRNA1-intron, shRNA2 or shRNA2-intron vector. The total cell number and the viability were normalized by the values for cells transfected with the control vector and are expressed as the mean ± S.D. of the percent of control. (D) The efficiency of the shRNA and shRNA interrupted by the intron was also analyzed by transiently transfecting 293T cells and using the Dual-luciferase Reporter Assay System.

We first constructed two vectors in which the shRNA with the miRNA-derived loop was inserted downstream of the CMV promoter. The first shRNA (shRNA1) contained 25 bases of an antisense strand targeting bases 851-875 of the firefly luciferase transcript. The second shRNA (shRNA2) contained 19 bases of the antisense strand targeting bases 155-173 of the firefly luciferase transcript, and it was followed by a poly (U) termination signal based on a report that the poly (U) termination signal could substitute for the polyadenylation signal in the expression of shRNA under the CMV promoter [20]. The efficiency of target silencing was then analyzed by transiently transfecting the shRNA vector in Cos cells with two vectors expressing firefly luciferase and *Renilla *luciferase, and luciferase activity was analyzed 48 h and 60 h after transfection (Figure 2A). In both cases, the reduction of firefly luciferase activity relative to the control vector indicated the efficiency of the shRNAs. In the case of shRNA2, the addition of the poly (U) termination signal appeared to be effective for target silencing because the construct without the poly (U) termination signal (siRNA2-ΔU) produced less efficient target silencing (Figure 2A).

### Interruption of shRNA-mediated target suppression by an intron with cell-type specificity

The loop derived from hsa-mir-371 was sufficient for shRNA-expressing vectors to induce RNAi; therefore, a group I intron was inserted into the middle site of the loop sequence (Figure 1B). When the constructed vectors were transiently cotransfected in Cos cells, we observed decreased firefly luciferase activity compared to control vectors (Figure 2B). No significant changes in the shape or viability of the transfected cells were observed (Figure 2C). Furthermore, it is not likely that the group I intron induced the silencing effect via a mechanism such as trans-splicing, that is, direct association before splicing. The sequence corresponding to the 5' exon has been used as antisense, and relatively long sequences have been used as the antisense strand for efficient splicing in trans-splicing systems [21]. In the present study, the antisense strand corresponds to the 3' stem of both shRNA1 and shRNA2. Moreover, if it were transcribed, the antisense strand would be followed by an unrelated sequence, specifically the poly (A) tail in the case of the shRNA1-intron and the poly (U) termination signal in the shRNA2-intron. We also repeated this experiment using another routinely used cell line, 293T cells. In stark contrast to the Cos cells, we found no significant repression of luciferase activity, but a similar or even greater silencing effect was observed when the shRNAs without the groupI intron were expressed (Figure 2D). In addition, we observed no significant effect on cell viability (Figure 2C). These findings suggest that the suppressive potency of the shRNA-intron is not due to direct activity by the RNase III enzyme DICER and that the splicing efficiency appears to affect the extent of luciferase gene silencing (Figure 2D).

**Figure 3:**
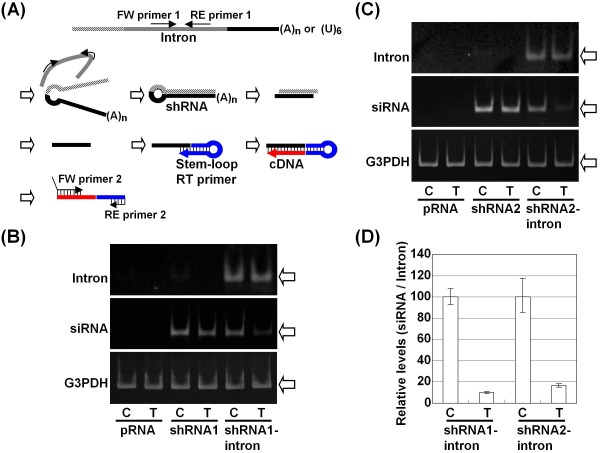
**Characterization of the processing of the shRNA-intron in Cos and 293T cells**. (A) The predicted processing of the shRNA-intron *in vivo *as well as the primers and a stem-loop primer are schematically illustrated. Primer 1 detected the unspliced mRNA and a spliced out intron, and primer 2 detected the siRNA. (B) The presence of siRNA and the intron was examined by end-point RT-PCR 48 h after the transfection of Cos cells (C) and 293T cells (T) with vectors expressing shRNA1 or shRNA1-intron. The empty pRNA-CMV3.1-Neo vector was used as a control. (C) The siRNA levels detected in cells transfected with the shRNA2 and shRNA2-intron vectors. (D) The levels of siRNA compared to those of the intron produced from the shRNA1-intron or shRNA2-intron vector in Cos cells and 293T cells were analyzed by RT-qPCR (each normalized to G3PDH mRNA). The normalized values of the siRNA/intron levels were set to 100 in Cos cells.

To elaborate on the difference between cell-types, we assessed the expression level of the siRNAs from the shRNA and shRNA-intron vectors. To this end, we designed a stem-loop primer to detect the predicted siRNA and determined that the siRNA was processed from the shRNA precisely as predicted (Figure 3A). We did not observe a significant difference in siRNA expression between Cos cells and 293T cells when the same amount of the shRNA-expressing vector was transfected (Figure 3B, C). However, the amount of siRNAs produced from the shRNA-intron vectors was significantly reduced in 293T cells compared with Cos cells, although the expression level of the intron was not significantly different between the cell types (Figure 3B, C). When we compared the siRNA to intron ratio, as assessed by RT-qPCR, in 293T cells and Cos cells, we confirmed a significant reduction in the level of siRNA (Figure 3D). Taken together, these results suggest that the splicing event and the resulting RNAi appear to occur in a cell-type specific manner, independent of the DICER activity of the cell.

### Characterization of sequence-dependent target silencing induced by the shRNA-intron

**Figure 4:**
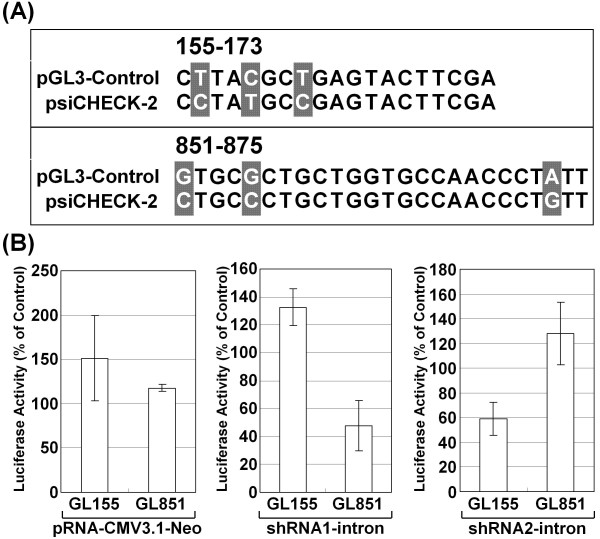
**Sequence-specific silencing effect of the shRNA-intron**. (A) The firefly luciferase sequences in the pGL3-Control vector that correspond to the targeting regions of 155-173 and 851-875 have a slightly different sequence composition than those in the psiCHECK-2 vector. The sequences from the pGL3-Control plasmid were synthesized and cloned downstream from the stop codon of the *Renilla *luciferase gene in the psiCHECK-2 vector. (B) The sequence-specific repression by shRNA interrupted by an intron was analyzed by transient cotransfection into Cos cells with the GL155 or GL851 vector. The empty pRNA-CMV3.1-Neo vector was used as a control. Luciferase activity was assessed using the Dual-luciferase Reporter Assay System, in which *Renilla *luciferase activity was normalized to firefly luciferase activity, and the data are shown as the mean ± S.D. of the percentage of the value for the empty psiCHECK-2 vector.

To more precisely evaluate whether the observed suppressive effects were specific for the target sequences, we used the psiCHECK-2 vector, which is typically used to check the knockdown efficiency of a target sequence by siRNA or miRNA [22]. Although the psiCHECK-2 vector expresses firefly luciferase in addition to *Renilla *luciferase, the firefly luciferase gene in this vector has a different sequence from the "155-173" and "851-875" target sequences of the pGL3-Control vector (Figure 4A). Even under this circumstance, it is plausible that these corresponding sites of psiCHECK-2 vector are also repressed by miRNA interference because unlike siRNA, miRNA does not require perfect complementarity of sequences. Based on the premise that miRNA commonly targets the 3' untranslated region of mRNA and that the silencing effect of siRNA is usually stronger than that of miRNA, the experimental readout would largely reflect the silencing induced by the siRNA [23]. Therefore, we constructed vectors in which the target sequence for each of shRNA1 and shRNA2 was inserted into the 3' UTR of the *Renilla *luciferase gene of the psiCHECK-2 vector. The constructed vectors, "GL851," which would be targeted by shRNA1, and "GL155", which would be targeted by shRNA2, were transfected, and we assessed the effect of each shRNA-intron on luciferase activity in the transfected cells. As expected, we observed a specific knockdown that corresponded to the inserted target sequences (Figure 4B). Based on these findings, it is plausible that the siRNA was produced from the shRNA-intron and silenced the target in a sequence specific manner.

### The modulation of splicing efficiency by an aptamer-ligand interaction

**Figure 5:**
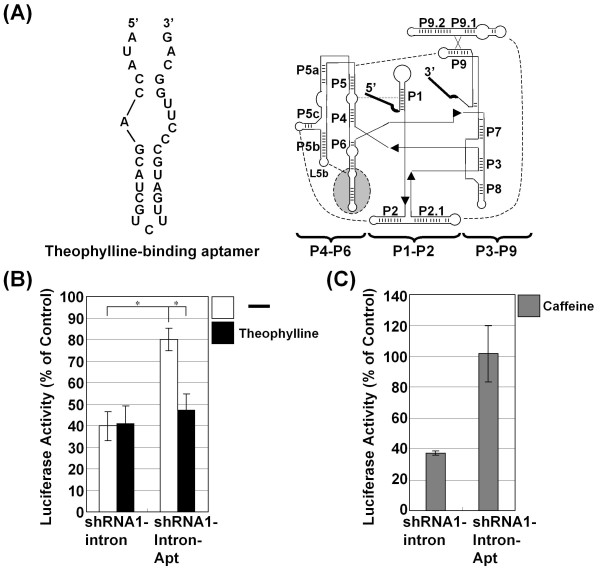
**Characterization of the shRNA-intron fused to a theophylline-binding aptamer**. The theophylline-binding aptamer is shown on the left. A schematic representation of the shRNA1-intron construct is shown on the right. The shRNA1-intron-Apt was constructed by substituting the region of the P6 stem, highlighted by a gray-shaded ellipse with a broken line, with the theophylline-binding aptamer in an analogous fashion to the construct "Th2P6" of Thompson, et al. (2002). In the diagram, straight lines are used to connect the three structural domains (labeled P4-P6, P1-P2 and P3-P9). P stands for the paired region. The arrowheads on the lines indicate 5' to 3' polarity. The dotted lines indicate tertiary interactions to help in correct folding. (B) Cotransfection of the shRNA1-intron-Apt into Cos cells along with vectors expressing firefly luciferase and *Renilla *luciferase was performed. The medium was removed and exchanged for fresh medium with or without 10 mM theophylline 24 h after transfection. Thereafter, the plates were incubated for 6 h and medium was replaced with normal, fresh medium. Luciferase activity was analyzed 36 h after transfection. (C) Caffeine treatment at a concentration of 10 mM was performed in the same way as theophylline. The results are expressed as the mean ± S.E. of the percentage of the value for the control vector, with significance determined by t tests shown by *P < 0.05.

We assumed that the expression of shRNA from the shRNA-intron is modulated by a cell specific factor at the step at which the inserted intron in the loop region of the shRNA is spliced. Considering that the regional binding of a chaperone protein could promote self-splicing *in vivo*, we set out to elucidate the effect of a potential trans-effector that binds the regional site in the group I intron in cultured mammalian cells. Intriguingly, the splicing of an aptamer-appended T4 phage group I intron was shown to be modulated in *E. coli *[13]. To accomplish aptamer-mediated targeting, the P6 region of the *Tetrahymena *group I intron was replaced with a theophylline-binding aptamer (Figure 5A). The activity of the shRNA-intron-aptamer vector was validated in Cos cells. The substitution of the theophylline-binding aptamer for the P6 region of the shRNA1-intron led to less efficient silencing, prompting us to investigate the activity of the shRNA1-intron-aptamer when the ligand for the aptamer was present in the culture medium (Figure 5B). Consistent with previous reports of the use of an anti-theophylline aptamer in mammalian cells, the shRNA1-intron-aptamer showed a prominent effect with 8-10 mM theophylline [24,25]. This concentration of theophylline, however, was highly toxic to the cultured cells. Therefore, we decreased the exposure period and observed a repressive effect similar to that of the shRNA1-intron. The presence of theophylline in the culture medium had no effect on the silencing activity induced by the shRNA1 intron vector (Figure 5B). Adding caffeine (10 mM), which is structurally similar to theophylline but does not bind to the aptamer, did not affect the silencing efficiency (Figure 5C) [26]. Hence, the aptamer-appended group I intron became functional by adding theophylline in a manner analogous to that of the T4 phage group I intron in *E. coli *[13]. The similar effect, although slightly less robust relative to that of the T4 phage group I intron in *E. coli*, might be due to the differences in the physiologic conditions between mammalian cells and *E. coli *and in their structure. Nevertheless, we obtained evidence that the intracellular milieu affects the splicing efficiency of different cell lines (Figure 2).

## Discussion

The efficient splicing of group I introns *in vivo *has been attributed mainly to chaperone proteins, contrasting with splicing *in vitro*, which occurs in the absence of any proteins [12,27]. In fact, compared to Cos cells, very little target silencing by shRNA-intron vectors was observed in 293T cells, despite the fact that the corresponding shRNA expression vectors had the same or even better effects on target silencing. Given these results, target silencing by shRNA-intron vectors might be dependent on the endogenous distribution or the amount of chaperone proteins in different cell types. Alternatively, some substrate-specific chaperone proteins or some inhibitory factors might be involved in the splicing reaction [28-30]. From a therapeutic point of view, it is intriguing that a particular chaperone protein is overexpressed in cancer cells and that could be considered a target for cancer therapy [31]. There is evidence that considerable splicing occurs in 293 cells when group I introns are inserted into a luciferase gene [2]. Therefore, there might be a preferential length, sequence, and structure for *in vivo *splicing [32]. Thus, several features and obstacles of intron self-splicing are evident when producing shRNA via splicing to analyze RNA chaperone activity in mammalian cells, similar to an "RNA folding trap" [6,33].

The *Neurospora crassa *mitochondrial tyrosyl-tRNA synthetase (CYT-18 protein) binds the P4-P6 domain of the *Tetrahymena *group I intron to promote splicing [34]. However this effect has been confirmed only for the synthetases of *Neurospora crassa *and the closely related fungus *Podospora anserina*, despite the fact that these proteins share the same basic structure with all bacterial tyrosyl-tRNA synthetases and the *Saccharomyces cerevisiae *mitochondrial tyrosyl-tRNA synthetase. Theophylline, a small organic molecule, modulates group I intron activity in *E. coli *and in mammalian cells, and there is a possibility that such a protein could bind and trigger a change in the enzymatic activity of the intron in these species. However, replacement of the P6 region with a theophylline-binding aptamer was not sufficient to completely inactivate self-splicing in Cos cells. Therefore, further replacement or modification could be rationally engineered for finely tuned control. Alternatively, antibiotics, which specifically bind to group I introns *in vitro*, might also modulate self-splicing; however, many of these compounds remain to be validated *in vivo*, and others are not as efficient as initially expected [35-39]. Altogether, further understanding of the splicing efficiency of group I introns in physiological conditions might pave the way for applications using modified group I introns and functional RNAs *in vivo *[40].

## Conclusions

Our results revealed that a miRNA-derived loop could be used to efficiently express shRNAs under a CMV promoter and that the expression of the shRNAs could be mediated by a group I intron inserted into the loop region. The shRNAs expressed via self-splicing of a group I intron affected target silencing in a cell-type specific manner under physiological conditions. In addition, the effect of theophylline when a theophylline-binding aptamer was embedded in a group I intron supported the feasibility of regulation by a trans-effector as well as the physiological importance of the connection between splicing efficiency and cellular factors.

## Methods

### Plasmid construction

pRNA-CMV3.1-Neo (GenScript Corporation) was used to construct the vectors expressing shRNA and shRNA interrupted by an intron. For the shRNA vectors, a pRNA-CMV3.1-Neo vector was digested with BamHI and HindIII and was ligated to the annealed oligonucleotide. The oligonucleotides used were as follows: shRNA1: 5'-GATCCGTGCGCTGCTGGTGCCAA CCCTATTTTTCTGCCCTCTTGTGAAAAATAGGGTTGGCACCAGCAGCGCACA-3' and 5'-AGCTTGTGCGCTGCTGGTGCCAACCCTATTTTTCACAAGAGGGCAGAAAAATAGGGTTGGCACCAGCAGCGCACG-3'; and shRNA2: 5'-GATCCTTACGCTGAGTACTTCGAT TTCTGCTCTCTTGTGAAATCGAAGTACTCAGCGTAAGTTTTTTGGAA-3' and 5'-AGCT TTCCAAAAAACTTACGCTGAGTACTTCGATTTCACAAGAGAGCAGAAATCGAAGTACTCAGCGTAAG-3'. These vectors were digested with BamHI, treated with mung bean nuclease to remove 5' extensions, and self-ligated. To construct the shRNA2 vector, a one base modification of its loop region was performed using the KOD-Plus-Mutagenesis Kit (TOYOBO) according to the manufacturer's instructions. The following primers were used: forward primer: 5'-CCCTCTTGTGAAATCGAAGTACTC-3' and reverse primer: 5'- CAGAAATCGAAGTA CTCAGC-3'. The pTZISVU vector, which contains the group I intron from *Tetrahymena thermophila*, was digested with EcoRI and HindIII, and its intron-containing fragment was subcloned into the EcoRI-HindIII restriction site of pVAX1 (Invitrogen) to generate pVAX1-intron vector. To construct the shRNA1-intron vector, a vector containing the following sequence at the BamHI-HindIII site of the pRNA-CMV3.1-Neo vector was first constructed: 5'GATCCGTGCGCTGCTGGTGCCAACCCTATTTTTCTGCCCTCTAAATAGCA AGTATTTTTCACGTGTGAAAAATAGGGTTGGCACCAGCAGCGCACA-3'. This vector (preshRNA1-intron) was then digested with PmlI, and the PCR-amplified intron was ligated. The following primers were used: forward primer: 5'-TGGAGGGAAAAGTTATCAGGC-3' and reverse primer: 5'-GAGTACTCCAAAACTAATCAATATACT-3'. To construct the vector expressing the shRNA2-intron, the vector containing the following sequence at the BamHI-HindIII site of the pRNA-CMV3.1-Neo vector was first constructed: 5'-GATCCTTACGCTGAGTACTTCGATTTCTGCCCTCTAAATAGCAATATT TTTCACGTGTGAAATCGAAGTACTCAGCGTAAGTTTTTTGGAA-3'. This vector (preshRNA2-intron) was digested with PmlI, and the PCR-amplified intron was ligated as well in the shRNA1-intron. To construct the shRNA1-intron-Apt vector, the following oligonucleotides were annealed and ligated into the pVAX1-intron digested with AhdI and EcoNI: 5'-TAGTCTGTGAACTGCATCCATATCCTGCCAAGGGCATCAAGACGATGCT GGTATGACTTGGCTGCGTGGTTAGGACCATGTCCGTCAGCTTATTACCATACCCTTT-3' and 5'-CAAAGGGTATGGTAATAAGCTGACGGACATGGTCCTAACCACGCAGCCAA GTCATACCAGCATCGTCTTGATGCCCTTGGCAGGATATGGATGCAGTTCACAGACTAA-3'. The resulting vector was PCR-amplified with the following primers then ligated with the preshRNA1-intron digested with PmlI: forward primer: 5'-TGGAGGGAAAAGTTATCAGG C-3' and reverse primer: 5'-GAGTACTCCAAAACTAATCAATATACT-3'. The GL851 and GL155 vectors were constructed by inserting the annealed oligonucleotide into the PmeI site of the psiCHECK-2 Vector (Promega). All subcloned sequences were verified by DNA sequencing.

### Cell culture and transfections

Cos cells and 293T cells were maintained in Dulbecco's modified Eagle's medium (DMEM) supplemented with 10% fetal bovine serum, 100 U/ml penicillin, and 100 μg/ml streptomycin in a 5% CO2-humidified incubator at 37°C. One day before transfection, the cells were trypsinized and seeded into 24-well plates at a density of 2-3 × 10^4 ^cells/well. Cotransfection was performed using FuGENE6 (Roche Diagnostics) according to the manufacturer's instructions. Specifically, 1 ng of pGL3-Control vector (Promega) and 5 ng of phRG-TK vector (Promega) for Cos cells or 5 ng of pGL3-Control vector and 1 ng of phRG-TK vector for 293T cells were cotransfected with 400 ng of the shRNA vector, shRNA-intron vector, or shRNA-intron-Apt vector using 1 μl of the FuGENE6 reagent per well. For the theophylline or caffeine treatment, the cells were incubated in a 5% CO2-humidified incubator at 37°C in DMEM supplemented with 10 mM theophylline or caffeine beginning 24 h after transfection. After 6 h, the medium containing theophylline or caffeine was again exchanged to normal DMEM. After incubation with cell culture medium for the indicated times, the cells were lysed in a passive lysis buffer (Promega). Firefly and *Renilla *luciferase signals were measured using the Dual-Luciferase^® ^Reporter Assay System (Promega).

### Determination of cell number and viability

A total of 2 × 10^4 ^cells/well of Cos cells and 3 × 10^4 ^cells/well of 293T cells were seeded into 24-well plates. After incubation for 24 h, 400 ng of the vector, shRNA1, shRNA1-intron, shRNA2 or shRNA2-intron was transfected. At the same time, the cells were also transfected with the pRNA-CMV3.1-Neo empty vector as a control. After incubation for 48 h and 60 h, the total cell number and viability were determined using a standard trypan blue membrane permeability assay. Live and dead cells were stained with trypan blue, the total cell numbers were counted on a conventional hemocytometer, and the percent viability was calculated as the number of live cells/total number of cells × 100. The total cell number and the viability of the cells transfected with the control vector were set at 100%. The number and viability of each type of transfected cell are expressed as the mean ± S.D. of the percent of control.

### RNA extraction, reverse transcription (RT), and real-time qPCR

Total RNA samples from Cos cells and 293T cells were obtained 48 h after transfection using the mirVana miRNA Isolation Kit (Ambion) according to the manufacturer's protocol. For detection of siRNAs, stem-loop RT primers were designed, and pulsed reverse transcription was performed [41,42]. The stem-loop RT primer for the shRNA1 and shRNA1-intron had the following sequence: 5'-GTCGTATCCAGTGCAGGGTCCGAGGTATTCGCACTGGATACG ACGCTGCT-3'. The sequence for the shRNA2 and shRNA2-intron stem-loop RT primer was as follows: 5'-GTCGTATCCAGTGCAGGGTCCGAGGTATTCGCACTGGATACGACAACTT A-3'. For detection of the intron and G3PDH mRNA, cDNAs were synthesized with the ReverTra Ace qPCR RT Kit (TOYOBO). qPCR analysis was performed using specific primer pairs and the THUNDERBIRD qPCR Mix (TOYOBO). The results were evaluated by the comparative threshold cycle method [43]. The following primers were used: for the siRNAs produced from the shRNA1 and shRNA1-intron vectors, forward primer: 5'-TCGCGAATAGG GTTGGCACC-3' and reverse primer: 5'-GTGCAGGGTCCGAGGT-3'; for the siRNAs produced from the shRNA2 and shRNA2-intron vectors, forward primer: 5'-TCGCGTCGAAG TACTCAGCG-3' and reverse primer: 5'-GTGCAGGGTCCGAGGT-3'; for the intron, forward primer: 5'-GCCTTGCAAAGGGTATGGTAAT-3' and reverse primer: 5'-TAGGACTTGGCTG CGTGGTT-3'; and for G3PDH, forward primer: 5'-AACAGCGACACCCACTCCTC-3' and reverse primer: 5'-TCCACCACCCTGTTGCTGTA-3'. The end-point PCR products were electrophoresed on a 15% polyacrylamide gel and were stained in ethidium bromide.

## Authors' contributions

KN designed and conducted most of the experimental work, compiled and analyzed the data, and drafted the manuscript. YI participated in constructing the expression vectors and in conducting the cotransfection experiments. HT provided funding and finalized the manuscript. All authors approved the final manuscript.
